# SOX9 transduction of a human chondrocytic cell line identifies novel genes regulated in primary human chondrocytes and in osteoarthritis

**DOI:** 10.1186/ar2311

**Published:** 2007-10-12

**Authors:** Simon R Tew, Peter D Clegg, Christopher J Brew, Colette M Redmond, Timothy E Hardingham

**Affiliations:** 1 UK Centre for Tissue Engineering, Wellcome Trust Centre for Cell-Matrix Research, Faculty of Life Sciences, University of Manchester, Michael Smith Building, Oxford Road, Manchester M13 9PT, UK; 2 Faculty of Veterinary Sciences, University of Liverpool, Leahurst, Neston, CH64 7TE, UK

## Abstract

The transcription factor SOX9 is important in maintaining the chondrocyte phenotype. To identify novel genes regulated by SOX9 we investigated changes in gene expression by microarray analysis following retroviral transduction with *SOX9 *of a human chondrocytic cell line (SW1353). From the results the expression of a group of genes (*SRPX*, *S100A1*, *APOD*, *RGC32*, *CRTL1*, *MYBPH*, *CRLF1 *and *SPINT1*) was evaluated further in human articular chondrocytes (HACs). First, the same genes were investigated in primary cultures of HACs following *SOX9 *transduction, and four were found to be similarly regulated (*SRPX*, *APOD*, *CRTL1 *and *S100A1*). Second, during dedifferentiation of HACs by passage in monolayer cell culture, during which the expression of SOX9 progressively decreased, four of the genes (*S100A1*, *RGC32*, *CRTL1 *and *SPINT1*) also decreased in their expression. Third, in samples of osteoarthritic (OA) cartilage, which had decreased *SOX9 *expression compared with age-matched controls, there was decreased expression of *SRPX, APOD, RGC32, CRTL1 *and *SPINT1*. The results showed that a group of genes identified as being upregulated by SOX9 in the initial SW1353 screen were also regulated in expression in healthy and OA cartilage. Other genes initially identified were differently expressed in isolated OA chondrocytes and their expression was unrelated to changes in SOX9. The results thus identified some genes whose expression appeared to be linked to SOX9 expression in isolated chondrocytes and were also altered during cartilage degeneration in osteoarthritis.

## Introduction

The chondrocytes within articular cartilage are responsible for the maintenance of the specialized extracellular matrix (ECM) of the tissue and for its biomechanical properties. The chondrocyte phenotype is characterized by the expression of specific genes, such as collagen type II and the transcription factor *SOX9 *[1]. Collagen type II is an abundant component in the cartilage ECM and is essential for its integrity. Damage to collagen type II and loss of other cartilage ECM components occur during degenerative joint diseases such as osteoarthritis (OA), which result in severe disability and present a major health problem in the ageing population [2]. This may arise from complex pathogenic mechanisms, which result in decreased matrix synthesis and upregulated pathways of tissue degradation [3]. Characteristic of cartilage in OA are changes in the expression of ECM genes and the downregulation of the key chondrogenic transcription factor *SOX9 *[4,5].

A large number of cartilage matrix genes have been shown to come under the transcriptional control of *SOX9*. They include *COL2A1*, *COL9A1*, *COL11A2*, aggrecan and cartilage link protein (*CRTL1*) genes [6-9], all of which play important roles in articular cartilage structure and function. Furthermore, *SOX9 *is expressed in presumptive cartilage during embryo development, and mutations in the human *SOX9 *gene, which result in haploinsufficiency of SOX9, cause campomelic dysplasia with skeletal malformation and dwarfism [10]. Moreover, mice chimaeras containing both wild-type and *SOX9*-null cells develop normally, but there is no contribution by the *SOX9*-null cells towards cartilage formation [11].

The expression of *SOX9 *declines rapidly in chondrocytes that are isolated and cultured in monolayer [12], and this is accompanied by a reduction in the expression of cartilage matrix genes such as *COL2A1 *[13]. Overexpression of *SOX9 *in human chondrocytes passaged in culture increases *COL2A1 *expression and increases their capacity to reform a cartilage ECM when placed in chondrogenic culture [14-16]. In view of the importance of *SOX9 *in the development and maintenance of the chondrocyte phenotype, its down regulation in OA is clearly likely to contribute to cartilage pathology. We investigated *SOX9 *transduction of a human chondrocytic cell line to identify genes that are differentially expressed in the presence by *SOX9*. We then investigated the expression of these genes in both cDNA samples representative of normal or OA cartilage and in primary human articular chondrocytes during culture and dedifferentiation to establish whether they were similarly regulated *in vitro *and in cartilage pathology.

## Materials and methods

### Tissue collection

Osteoarthritic cartilage was obtained from patients undergoing total knee arthroplasty for clinically and radiologically diagnosed OA [17]. Patients were excluded if there was any history of inflammatory arthropathies, or infection within the knee. Normal articular cartilage was obtained from patients undergoing above-knee amputation who had no history of joint disease. All tissue was obtained with fully informed consent and ethical approval. For tissue culture, cartilage from intact regions of joints with clinical confirmation of degenerative OA was harvested and subject to sequential trypsin/collagenase digestion to isolate chondrocytes as previously described [14]. For gene-expression studies, paired full depth samples were taken from each joint (8 normal joints and 15 OA joints), with one sample being harvested from a major loaded area on the medial femoral condyle (MFC), and one from the less loaded lateral posterior condyle (LPC), placed in RNAlater and transferred to the laboratory on ice.

### Culture and retroviral transduction of cells

Monolayer cultures of SW1353 cells were kept in Dulbecco's modified Eagles medium (DMEM) supplemented with 10% foetal bovine serum (FBS), 100 units/ml penicillin and 100 units/ml streptomycin (all from Cambrex, Wokingham, UK) at 37°C, 5% CO_2_. For retroviral transductions, 40% confluent cultures were infected in standard culture medium with an RKAT retrovirus containing a bicistronically expressed cDNA encoding human FLAG tagged *SOX9 *and green fluorescent protein (*GFP*), at a titre of 5 × 10^6 ^[14]. After three repeated transductions, more than 90% of the cells were transduced and the cells were designated SOX9-SW1353. Cells transduced with a *GFP*-only retrovirus were used as controls and designated GFP-SW1353. SOX9 protein was assessed in the cells by immunoblotting using an anti-SOX9 goat polyclonal antibody (H-90, Santa Cruz Biotechnology, Calne UK). Human articular chondrocytes were isolated from cartilage on OA knee joints and maintained in culture in medium (as above) [14]. Cells were harvested for gene-expression analysis within the first week of culture (P0) and after 1 and 2 passages (P1 and P2). HACs were transduced with SOX9 or GFP-only retrovirus at passage 4 after first increasing their proliferation rate by adding platelet derived growth factor BB, transforming growth factor β1 and fibroblast growth factor 2 to the culture medium [14]. Gene expression in these cells was analysed at passage 6–8.

### Microarray analysis

Glass spotted microarrays (Human known gene SGC oligo set array number 1) were obtained from the Human Genome Mapping Project. Each glass slide contains 9600 spotted oligonucleotides printed in duplicate, approximately 600 bp in length corresponding to the 3' region of each gene's mRNA. Probes were created from RNA, which was isolated from confluent monolayer cultures of GFP-SW1353 or SOX9-SW1353 using Tri Reagent (Sigma, Poole UK). 50 μg of total RNA was added to 2 μg of oligo d(T)_16 _(Invitrogen, Paisley, UK) and incubated at 70°C for 10 minutes before snap cooling on ice for 2 minutes. RNA was reverse transcribed to produce a cDNA probe in a labelling mix containing 1× first strand synthesis buffer, 500 μM DTT, 500 μM dATP, 500 μM dTTP, 500 μM dGTP, 100 μM dCTP, 400 units of superscript II reverse transcriptase (Invitrogen) and either 100 μM Cy3–dCTP or 100 μM Cy5–dCTP (Amersham, Uxbridge, UK). Labelling reactions were incubated for 2 hours at 42°C before adding EDTA to 1 mM to stop the reaction. RNA in the samples was degraded by adding sodium hydroxide to 25 mM and heating at 70°C for 10 minutes. Samples were neutralised by addition of hydrochloric acid, and labelled cDNA was purified using a PCR clean up kit (Qiagen, Crawley UK). The purified probe was eluted in 50 μl of nuclease free water and combined with 10 μg of human Cot-1 DNA, 6 μg oligo d(A)_10–20_, and 3 μg oligo d(T)_16 _(all from Invitrogen), and the volume reduced to 18 μl by vacuum centrifugation. The probe was then combined with 18 μl of a 2× hybridisation solution (final concentration: 25% formamide, 5 × SSC and 0.1% SDS), boiled for 3 minutes and hybridised overnight at 42°C under a glass cover slip. The arrays were then washed for 3 minutes each in 2× SSC, 0.1× SSC/0.1% SDS, and 0.1× SSC. Raw intensities at 635 nm and 532 nm were obtained for analysis from four independently probed arrays using the same starting RNA sample using a GenePix 4000A confocal microarray scanner. This data was imported into MaxDView software [18] and each pair of red/green measurements were subjected to intensity-dependant normalization. This removed intensity-dependent bias introduced by the use of the two different fluorophores as probe labels using the Loess (Lowess) method [19] and converted the data to a log ratio with the mean set to zero following normalisation. Quadruplicate log ratios were averaged and standard deviations were determined. In addition, *t*-tests were carried out comparing the four replicates to zero to determine potentially significantly regulated genes. Data was filtered to display only those genes with *P *< 0.05 and greater than twofold change in expression. Raw data from each individual channel of each array was also subjected to principle components analysis (PCA) and hierarchical clustering following normalisation of data (log2, mean set to 0 and standard deviation set to 1) using Partek software. All pre-normalised data has been submitted to MIAMExpress [20] at the European Bioinformatics Institute to allow public access (ArrayExpress Accession number E-MEXP-826).

### RNA extraction and cDNA synthesis

#### Cell culture

Total RNA was prepared from monolayer SW1353 and HAC cultures using Tri Reagent. cDNA was synthesised from 1 μg of total RNA using M-MLV reverse transcriptase and primed with random hexamers oligonucleotides (Promega, Southampton, UK) in a 25 μl reaction.

#### Tissue extraction

Total RNA from cartilage was obtained by homogenization with Braun mikrodismembrator followed by Trizol extraction and chloroform/ethanol purification. Total RNA was then isolated using RNeasy minicolumns and reagents, according to the manufacturers instructions (Qiagen, Crawley, Surrey, UK) including on-column digestion of residual DNA using a RNase-Free DNAse kit (Qiagen) [21,22]. cDNA was synthesised from 10–100 ng of total RNA using global amplification methodology [23].

### Real time PCR analysis

Real time PCR was used to determine the expression of chondrocyte genes identified as being regulated by SOX9 in the microarray experiments. Amplification by PCR was carried out in 25 μl reaction volumes on a MJ Research Opticon 2 using reagents from a SYBR Green Core Kit (Eurogentec, Seraing, Belgium) with gene-specific primers designed using Applied Biosystems Primer Express software. Relative expression levels were normalised to GAPDH and calculated using the 2^-ΔCCt ^method [24]. Primer sequences for *GAPDH*, *COL1A1*, *COL2A1*, aggrecan, *SOX6*, and *SOX9 *for identification of the effect of *SOX9 *transduction in SW1353 have been described previously [15]. Primer sequences for the other genes of interest were designed with a 3' bias to allow accurate quantification of the globally amplified cartilage cDNA libraries (Table 1).

**Table 1:** Primer sequences used to quantify gene expression

Gene	Forward (5'-3')	Reverse (5'-3')
*GAPDH*	CACTCAGACCCCCACCACAC	GATACATGACAAGGTGCGGCT
*COMP*	CTGGGCCAACCTGCGTTA	CGCAGCTGATGGGTCTCATAG
*APOD*	ACGCCCTCGTGTACTCCTGTA	TTCCACAAGCACAAACTTTACACAT
*S100A1*	CCAGGAGTATGTGGTGCTTGTG	ATGTGGCTGTCTGCTCAACTGT
*RGC32*	GACAAAGACGTGCACTCAACCTT	ACTGTCTAAATTGCCCAGAAATGG
*SRPX*	TGGCTGGTTGATTTTGTAGAGAAA	TAGAAAAGAGTTAGGTGTCACATTGAATAA
*SPINT1*	CGAGTTGTTTCCTCGCTGATC	GCAATGGAATTCAACATAAGCAAA
*CRTL1*	TTCCACAAGCACAAACTTTACACAT	GTGAAACTGAGTTTTGTATAACCTCTCAGT
*CRLF1*	AACGGCCATAACAGCTCTGACT	ACTCAACCAACCCTCACACACA
*MYBPH*	AGGCCTACAGTCAAACTCCAGAGA	GAAGGGAGGCCAGCAGGTA

### Database analysis of conserved SOX9 binding regions

Candidate gene alignments were visualised using Vista Browser [25]. Conserved SOX9 consensus binding sites, defined by the Transfac database, were identified by comparing the human genome with that of mouse or dog using rVista [26]. *SPINT1 *and *GAPDH *genes were analysed in their entirety as well as up to 7 kb upstream of the transcription start site. Due to the very large intron size of the *APOD*, *RGC32 *and *SRPX *genes, only 7 kb upstream of the transcription start site and the regions within the first intron of these genes were included in this analysis. Conserved sites identified in both species comparisons were accepted as potential SOX9 binding regions.

### Statistical analysis

Unpaired *t*-tests were used to compare the effect of *SOX9 *transduction on cultured cells. Statistical analyses to identify the effect of both disease state and site within the joint on gene expression were performed using mixed effects linear regression models following transformation of the data to allow normal distribution.

## Results

### Changes in gene expression in *SOX9 *transduced SW1353 cells

**Figure 1:**
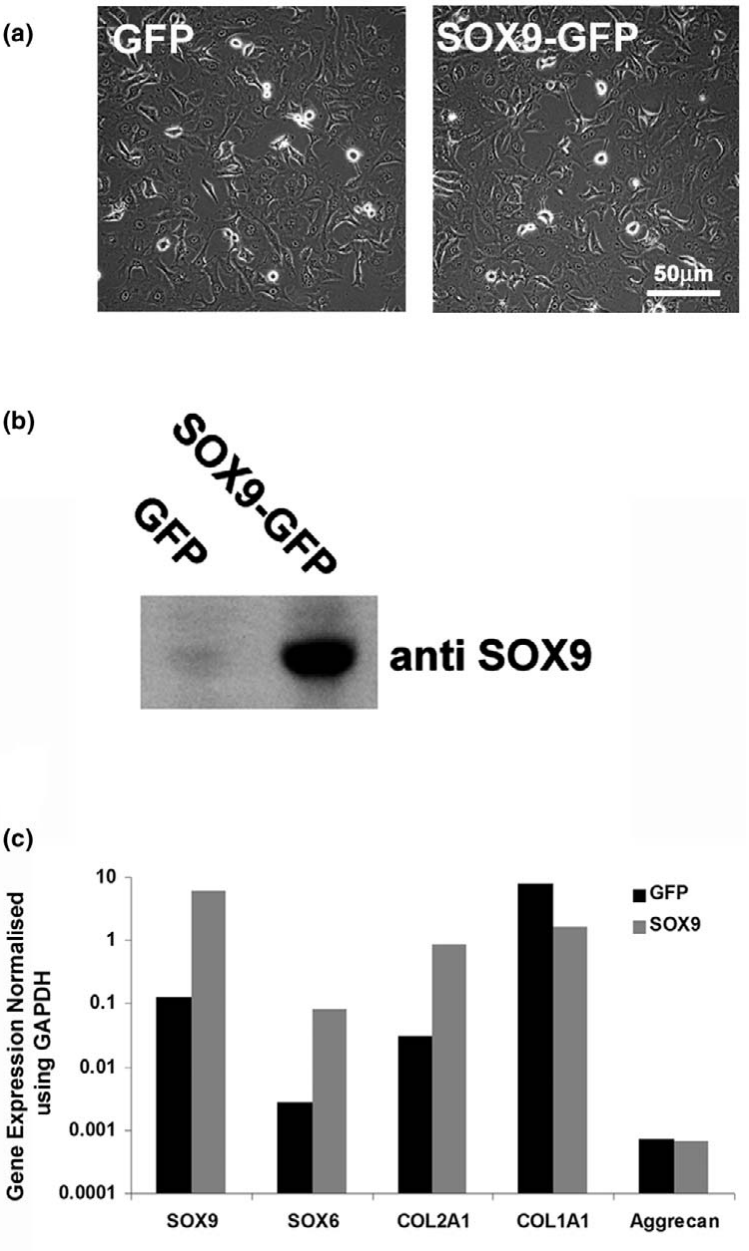
Retroviral expression of SOX9 in SW1353 chondrosarcoma cells. **(a)** Phase contrast micrograph demonstrating the morphology of SW1353 chondrosarcoma cells transduced with a retrovirus containing *GFP *or bicistronic *SOX9-GFP*. Scale Bar = 50 μm. **(b)** Cell lysates from *GFP*- or *SOX9*-SW1353 cells were analysed by western blotting using an anti-SOX9 antibody. **(c)** Real time PCR analysis of cDNA derived from green-fluorescent protein (GFP; black bars) or bicistronic *SOX9-GFP *(grey bars) transduced SW1353 chondrosarcoma cells.

Retroviral transduction with SOX9 was carried out on a human chondrocytic cell line (SW1353), which had previously been shown to have responses to growth factors and cytokines similar to primary chondrocytes [27] and provided RNA in amounts appropriate for microarray analysis. The SW1353 cells were transduced at ~90% efficiency with a *SOX9*-GFP bicistronic retroviral vector (*SOX9*-SW1353), and controls were transduced with a *SOX9*-free *GFP*-retrovirus (GFP-SW1353) (Figure 1a). The SW1353 cells were of interest for this study as their normal expression of SOX9 was much lower than human chondrocytes in cartilage (relative to GAPDH), whereas the level of SOX9 expression following transduction was increased by 18-fold (Figure 1c) and approached the level found in cartilage. There was no discernable change in morphology following SOX9 transduction. Immunoblotting confirmed that *SOX9*-SW1353 synthesised increased levels of the SOX9 protein compared with controls (Figure 1b). The cells also showed increased gene expression of *SOX6 *(up to 14-fold) and *COL2A1 *(up to 13-fold), but aggrecan expression was low and was unchanged by *SOX9*. The SW1353 cells expressed high levels of *COL1A1 *and this was reduced 6-fold by SOX9 transduction. The stimulation of both *SOX6 *and *COL2A1 *by *SOX9 *confirmed that SW1353 cells were responsive to this factor, unlike other non-chondrocytic cells, such as dermal fibroblasts, which failed to upregulate cartilage matrix genes in response to *SOX9 *transduction [28].

### Microarray analysis of SOX9-transduced SW1353 cells

Dual hybridisations were performed in quadruplicate (including duplicated orientations of dye to sample) using probes produced from single RNA samples from *SOX9*-SW1353 or *GFP*-SW1353 cells. Extensive filtering of the normalised data was carried out as described in the materials and methods section. From the original 9,600 different genes on each array, 22 were found to be upregulated and 9 were downregulated by *SOX9*. From these, eight of the most strongly regulated genes were selected for further analysis (Table 2).

**Table 2:** Candidate genes chosen following microarray analysis of SOX9 transduced SW1353 chondrosarcoma cells

Gene name	GenBank accession number	Fold upregulation	Fold downregulation
Apolipoprotein D (*APOD*)	NM_001647	22.89	NA
RGC32	NM_014059	10.82	NA
S100 calcium-binding protein A1 (*S100A1*)	NM_006271	8.84	NA
Sushi-repeat-containing protein X chromosome (*SRPX*)	NM_006307	3.47	NA
Cytokine receptor-like factor 1 (*CRLF1*)	NM_004750	3.31	NA
Cartilage linking protein 1 (*CRTL1*)	NM_001884	2.97	NA
Myosin-binding protein H (*MYBPH*)	NM_004997	NA	4.23
Kunitz-type protease inhibitor (*SPINT1*)	AF027205	NA	2.83

Genes demonstrated regulation consistently across quadruplicate microarray experiments. NA, not applicable.

### Real time PCR analysis of SOX9-regulated genes

Gene-expression analysis by quantitative real time (qRT) PCR was used to confirm the gene changes identified by the microarray analysis. Statistically significant upregulation was observed for *SRPX *(1.8-fold), *S100A1 *(7.9-fold), *APOD *(4.2-fold) *RGC32 *(2.3-fold) and *CRTL1 *(2 fold) with analyses from separately cultured SW1353 cells. Regulation of the expression of *SPINT1*, *CRLF1 *and *MYBPH *could not be confirmed.

Having previously shown that retroviral transduction with SOX9 of passaged human OA chondrocytes re-activated their potential to form cartilage matrix [15], we investigated the expression of the novel genes identified in SW1353 cells in human articular chondrocytes that had been expanded in monolayer culture and transduced with *SOX9*-retrovirus. The results showed significant upregulation (*P *< 0.05) of *S100A1 *(26.8-fold), *CRTL1 *(3.0-fold) and *SRPX *(1.7-fold) following *SOX9 *transduction. Interestingly, *SPINT1 *was also significantly upregulated in the chondrocytes (2.1-fold), which differed from the finding in the SW1353 cells. *APOD *was expressed at very low level in transduced and control human OA chondrocytes in monolayer culture, and although its expression appeared to be increased slightly following *SOX9 *transduction, no statistical analysis was possible. *MYBPH *expression was again unaffected by *SOX9*. Therefore, there were examples of genes that displayed similar responses to SOX9 transduction in both SW1353 cells and primary chondrocytes, but also genes for which there were clear regulatory differences between the cell types.

### Primary chondrocyte culture with decrease in SOX9 expression

**Figure 2:**
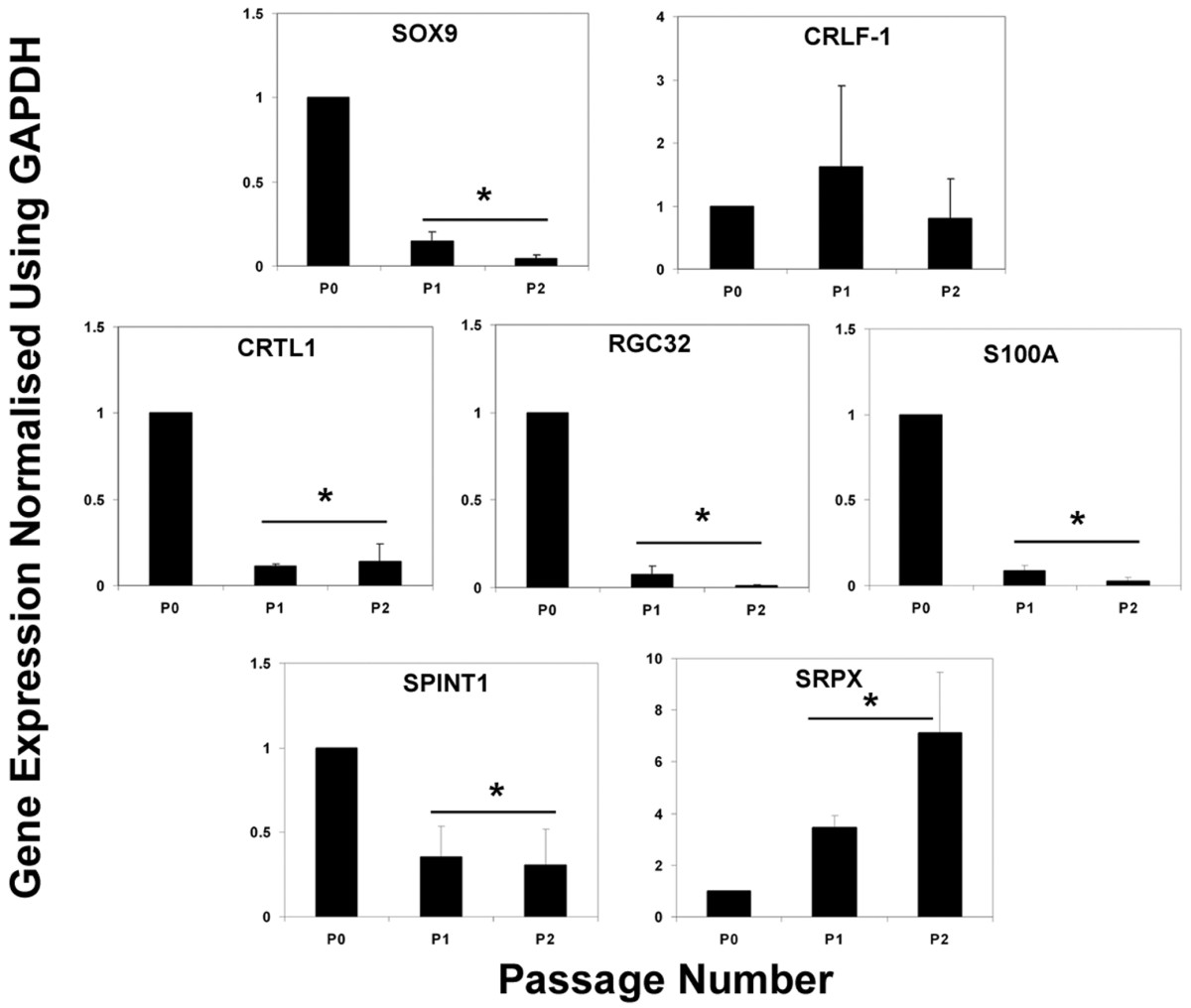
Regulation of candidate genes during chondrocyte dedifferentiation. Real time PCR analysis of candidate gene expression in cDNA from human articular chondrocytes at passage (P) 0, 1 or 2. Mean fold-change values (where P0 = 1) with standard errors are presented from chondrocytes cultures obtained from 3 donors. * indicates significant difference in expression compared with passage 0 levels *P *< 0.05 by paired students *t*-test.

To determine whether the expression of genes identified in this study were altered by non-viral-mediated changes in SOX9 expression, we investigated *in vitro *cultures of freshly isolated human articular chondrocytes (Figure 2). These cells were from OA cartilage, and had a lower expression of SOX9 in culture than in tissue, but still higher (relative to GAPDH) than in SW1353 cells. During monolayer culture of the OA chondrocytes there was a further 8–10-fold decrease in *SOX9 *expression, and we examined whether this was accompanied by any change in expression of the newly identified genes (Figure 2). A number of the genes including *S100A1*, *RGC32*, *CRTL1 *and *SPINT1 *were down regulated under these conditions, and therefore correlated with the reduction in *SOX9*. *SRPX*, in contrast, did not correlate with *SOX9 *in the monolayer cultured HAC, and its expression increased with passage. The expression of another gene, *CRLF1*, was unchanged during the fall in SOX9. The expression of *MYBPH *and *APOD *(one of the genes most strongly upregulated by *SOX9 *in SW1353 cells) were very low in these primary human articular chondrocytes, and significant regulation could not be identified.

### Expression of SOX9-regulated genes in normal and osteoarthritic cartilage

**Figure 3:**
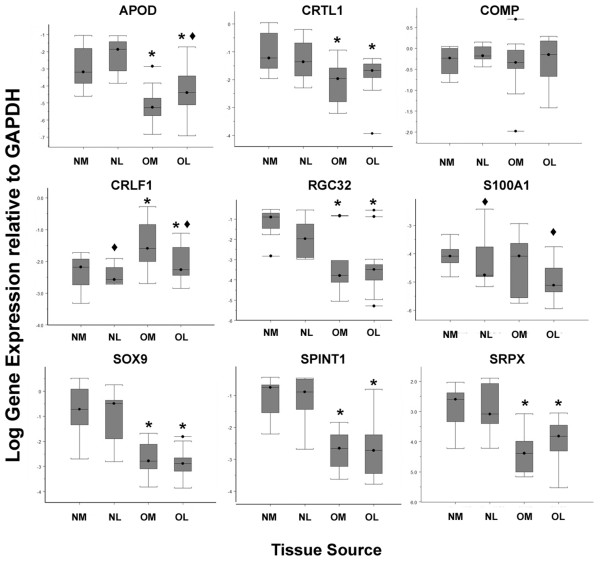
Comparison of the expression levels of candidate genes in normal and osteoarthritic cartilage. Real time PCR analysis of candidate gene expression in globally amplified cDNA representative of mRNA levels from normal (*n *= 8) or osteoarthritic (*n *= 15) human articular cartilage samples. Cartilage for the analysis was derived from either the medial or lateral femoral condyles. NM = normal medial, NL = normal lateral, OM = osteoarthritic medial and OL = osteoarthritic lateral. Symbols above bars indicate statistically significant regulation of that gene caused by:* disease (*P *< 0.05 mixed effects regression model) or ◆ joint location (*P *< 0.05 mixed effects regression model).

In a previous study [5] we showed that osteoarthritic cartilage consistently showed reduced expression of *SOX9 *compared with healthy age-matched control tissue. It was therefore of great interest to understand whether the newly identified genes were also altered in expression in OA cartilage. We therefore probed globally amplified cDNA samples from normal and OA femoral knee cartilage [5] for their expression (Figure 3). Furthermore, the tissues samples analysed were paired cartilage samples from high-load-bearing (MFC) and low-load-bearing regions (LPC) of the same joints. *SOX9 *gene expression was reduced (*P *< 0.0001) in the osteoarthritic samples compared with the age-matched controls, and there was no difference between differently loaded sites. Of the genes investigated, five were expressed at lower level in osteoarthritic cartilage (*CRTL1 *(*P *< 0.01), *SRPX *(*P *< 0.0001), *SPINT1 *(*P *< 0.0001), *RGC32 *(*P *< 0.001) and *APOD *(*P *< 0.0001)). One gene (*CRLF1*) was significantly upregulated in OA tissue compared with normal tissue (*P *< 0.01), and *S100A1 *showed a wide range of expression and no significant difference between OA and controls; however, analysis of the results from all the cartilage samples showed that its expression was correlated with SOX9. Most genes investigated showed similar expression in both the more highly loaded MFC and the lower loaded LPC sites. The exceptions to this were *APOD*, which was further reduced (*P *< 0.01) in expression in the more loaded and damaged cartilage, while both *S100A1 *(p < 0.03) and *CRLF1 *(p < 0.02) were expressed at higher levels in the more loaded tissue. The analysis of cartilage oligomeric matrix protein (*COMP*) gene expression showed that it was unaffected in OA (or location in the joint), and demonstrated that there was no generalised downregulation of all gene expression in OA chondrocytes.

### Genomic analysis of potential SOX9 binding sites in candidate genes

The candidate genes *SPINT1*, *SRPX*, *APOD, RGC32, CRTL1 *and *S100A1 *were among those whose expression followed that of SOX9 in most of the experimental systems that we examined. Of these genes, *CRTL1 *and *S100A1 *have previously been shown to possess SOX9 binding sequences within their promoter regions [9,29]. Potential SOX9 binding sites in non-coding, conserved regions of the genome in and around the other four gene loci were studied using rVista. This tool identifies conserved transcription factor binding sites in sequences based on homologies of such sites between different species, and in these genes it identified binding site conservation in human, mouse and dog sequences (Table 3). The analysis demonstrated conserved SOX9 binding regions in all four candidate genes. As a control, analysis of the house-keeping gene *GAPDH *revealed no potential SOX9 binding sites common to all three genomic sequences.

**Table 3:** Predicted SOX9 binding sites in candidate genes^a^

Gene	Conserved SOX9 binding site, relative to transcription start site	Transcription start site (bp position based on *homo sapiens *genome build 35.1)
*APOD*	+2998 bp to +3011 bp +3110 bp to +3123 bp	CHR3_RANDOM:544561
*GAPDH*	None passed criteria	CHR12:6513945
*RGC32*	+2534 bp to +2547 bp	CHR13:40929712
*SPINT1*	-1183 bp to -1170 bp	CHR15:38923534
*SRPX*	+3939 bp to +3952 bp +5628 bp to +5641 bp +15773 bp to +15786 bp	CHRX:37836348

^a^Base pair (bp) positions are given relative to the transcription start site of each gene. In all instances, positive numbers describe sites within the first intron of the gene.

## Discussion

The transcription factor SOX9 has been shown to control the transcription of a number of important cartilage matrix genes. It is able to interact with a conserved cartilage-specific enhancer element in the *COL2A1 *gene and can bind to promoter and enhancer regions in a number of other cartilage matrix genes [6-9]. This work has now identified a number of genes whose expression was changed in SW1353 cells by increasing SOX9 expression by retroviral transduction and may similarly contain conserved SOX9 response elements

The human SW1353 cells have previously been used to elucidate cytokine regulation of ECM-degrading proteases as model chondrocytes [27,30] and their *SOX9 *expression was shown to be increased by fibroblast growth factors 1, 2 and 9 and decreased by IL1β and TNFα [31]. They have also been used to identify cyclic AMP response element binding protein and p300 as novel partners of SOX9 that bind at cartilage-specific promoter sites [32]. Thus the SW1353 cells have some features of chondrocytes, but as with other chondrocytic cells in monolayer culture they expressed low levels of both cartilage ECM genes and SOX9, 6 and 5 [33]. Their transduction of cytokine signals has also been reported to differ from that seen in primary articular chondrocytes [33]. In this study SOX9-transduction increased the expression of target genes (such as *COL2A1*), although others appeared unaffected (such as aggrecan). The SW1353 cells therefore appear to lack some chondrocyte properties, but their response to SOX9 transduction was clearly more chondrocyte-like than dermal fibroblasts, which showed no regulation cartilage matrix genes in response to the overexpression of SOX9 [28].

From the initial microarray analysis we followed up gene-expression changes by qRT-PCR analysis in SOX9-SW1353 cells to confirm their regulation. Investigation of changes in the expression of this panel of genes in primary human chondrocytes following SOX9 transduction showed that some genes showed evidence of similar control to SW1353 cells, although some showed no comparable response. To extend these observations we investigated the expression in articular chondrocytes under conditions where the expression of SOX9 was changed by both natural and pathological factors. The expression was investigated in primary human chondrocytes cultured and passaged in monolayer, under which conditions the expression of SOX9 progressively becomes reduced. It was only after culture of the OA chondrocytes that the expression of SOX9 became reduced to the level found in the SW1353 cells before transduction. The change in expression during this fall in endogenous SOX9 expression showed *S100A1*, *RGC32*, *CRTL1 *and *SPINT1 *to decrease, which were therefore correlated with SOX9, as in SW1353 cells.

The identification of these SOX9-regulated genes led us to probe a human normal and OA cartilage library of globally amplified cDNA representing mRNA levels in chondrocytes in cartilage taken from load bearing or non-load-bearing regions from age-matched normal and OA human knees. *SOX9 *has been shown to be downregulated in osteoarthritis, and this may contribute to the pathological process by causing a reduction in the expression of ECM genes [4,5]. We found that many of the genes whose expression was altered by SOX9 in SW1353 cells and/or isolated primary chondrocytes displayed altered expression levels in OA cartilage (*CRTL1 *(*P *< 0.01), *SRPX *(*P *< 0.0001), *SPINT1 *(*P *< 0.0001), *RGC32 *(*P *< 0.001) and *APOD *(*P *< 0.0001)) compared with age-matched controls. It is worth noting that even a gene such as COL2A1, which is known to have SOX9 regulatory elements, has been demonstrated to poorly correlate with the expression of SOX9 in control and osteoarthritic cartilage [4], suggesting that in OA its expression is more dominantly controlled by other factors. It was therefore more interesting to identify genes such as *CRTL1*, *RGC32*, *S100A1 *and *APOD*, which had a pattern of expression closely correlating with SOX9 expression levels in SW1353 cells, in primary chondrocytes, and also in OA cartilage. *SRPX *generally correlated with SOX9, except during chondrocyte dedifferentiation, which may indicate that other factors predominantly influence it during this process.

*APOD*, which was expressed at relatively low levels in SW1353 cells, was expressed more strongly in cartilage, and the expression was reduced in OA, which was consistent with the decrease in *SOX9*. *APOD *encodes apolipoprotein D, which is a protein component of low density lipoprotein in human plasma [34], and is reported to be a transit protein in the skin [35]. It may therefore have some function in cartilage ECM. The finding that *APOD *is downregulated in OA agrees with two previous microarray studies comparing normal and OA tissue [36,37]. The present results showed further that *APOD *expression was not only downregulated in OA, but was also most strongly downregulated in the highly loaded, more physically damaged cartilage. *APOD *expression thus correlated with cartilage damage, whereas matrix genes, such as *CTRL1 *and *SOX9*, were similarly changed in OA in both low-loaded and high-loaded cartilage sites.

*S100A1 *encodes an intercellular calcium-binding protein, which can control myocardial contractility [38] and has recently been identified as an important SOX9 regulated gene that controls the terminal differentiation of chondrocytes [29]. *S100A1 *has previously been reported to be downregulated in osteoarthritis [36]. In the OA and control cartilage samples investigated here, *S100A1 *had lower mean expression in OA, but the difference was not statistically significant. However, its expression was found to be significantly correlated with *SOX9 *when the results from all cartilage samples were analysed (data not shown).

*SRPX *expression was increased by SOX9 transduction in SOX9-SW1353 and in primary human articular chondrocytes, and its expression was greatly reduced in OA cartilage. It therefore correlated well with SOX9 expression, although during chondrocyte dedifferentiation its expression increased more than sevenfold by passage 2 and was clearly unrelated to SOX9. This perhaps emphasises that any loss of chondrocyte phenotype in OA cartilage does not occur through a mechanism closely related to the loss of phenotype that occurs in these cells in monolayer culture. *SRPX *has a recognised role in ocular biology and disease. The *SRPX *gene encodes a putative membrane protein expressed abundantly in the retina, and was discovered as a candidate gene responsible for X-linked retinitis pigmentosa [39]. SOX9 has a potential regulatory role in the development of the retina, and may regulate the synthesis of collagen type II in the vitreous of the eye [40]. Furthermore, disrupted *SOX9 *expression in the 'odd sex' transgenic mouse, which results in sex reversal, also causes an eye phenotype with microphthalmia with cataracts [41]. The expression of SRPX may therefore be regulated by SOX9 during ocular development and may also have a role in cartilage biology.

Despite being unable to confirm any regulation by SOX9 in SW1353 by real time PCR, and with its expression unaffected in primary chondrocytes by the transition to monolayer culture, it was interesting that CRLF1 was significantly upregulated in osteoarthritic cartilage. CRLF1 protein is a member of the cytokine type I receptor family, and when expressed as a heterodimer with the cardiotrophin-like cytokine (CLC) can activate the membrane bound ciliary neurotrophic factor receptor-α (CNTFRα), which causes an interaction between gp130 and leukaemia inhibitory factor receptor, leading to cell signalling [42,43]. Further work characterising the expression of the genes encoding CNTFRα and CLC in cartilage is required as does the possibility that upregulation of *CRLF1 *expression could have a use as a marker of OA.

This study identified genes whose expression in chondrocytes was consistently correlated with changes in SOX9 expression. The results suggested that the expression of these genes may be regulated by SOX9, and as SOX9 is essential for chondrocyte phenotype, the novel genes with unknown function may help control the differentiated state of chondrocytes within cartilage. The correlation of expression with SOX9 linked these genes to changes in cartilage in OA. As OA is characterized by degenerative changes in cartilage it will be important to establish how the changes in the expression of SOX9-regulated genes contribute to the progressive loss of chondrocyte function and the compromise in cartilage integrity that occurs in OA.

## Conclusion

We have identified genes in a human chondrosarcoma cell line whose expression is altered by the overexpression of the chondrogenic transcription factor SOX9. Some of these genes were similarly regulated in primary human chondrocytes in response to changes in SOX9 induced by overexpression or by dedifferentiation in culture. The expression of some of these genes was also correlated with SOX9 expression in intact human articular cartilage, and was therefore suppressed in OA cartilage compared with age-matched control cartilage.

## Abbreviations

CLC = cardiotrophin-like cytokine; CNTFR = ciliary neurotrophic factor receptor; DMEM = Dulbecco's modified Eagle's medium; ECM = extracellular matrix; FBS = foetal bovine serum; GFP = green fluorescent protein; LPC = lateral posterior condyle; MFC = medial femoral condyle; OA = osteoarthritis.

## Competing interests

The authors declare that they have no competing interests.

## Authors' contributions

SRT conceived, designed and performed the experimental work associated with the microarray and was responsible for the initial versions of this manuscript. CJB collected the normal and OA cartilage and produced the cDNA libraries from femoral cartilage. CMR undertook the laboratory work associated with real time PCR analysis of the normal and OA cartilage libraries. PDC performed the statistical analyses, designed and validated the PCR primers, and supervised the project. TEH supervised and oversaw the completion of the studies as well as the writing of this manuscript. All authors read and approved the final manuscript.
